# LEVERAGING HIGHER EDUCATION IN OUR AGE-FRIENDLY WORLD

**DOI:** 10.1093/geroni/igae098.2941

**Published:** 2024-12-31

**Authors:** Kathy Black, Patricia Oh, Joann Montepare, Lenard Kaye

**Affiliations:** University of South Florida Sarasota-Manatee Campus, Sarasota, Florida, United States; University of Maine, Bangor, Maine, United States; Lasell University, Newton, Massachusetts, United States; University of Maine Center on Aging, Bangor, Maine, United States

## Abstract

There is increasing interest in better understanding the connection between higher education and age-friendly community efforts. The global age-friendly community (AFC) movement calls for multi-sectoral engagement in a multi-year model encompassing four core phases (engage, plan, act, measure) to improve livability in domains of community life pertaining to the built, social, and service environment. However, there is limited empirical knowledge regarding the involvement of higher education and how it supports AFC efforts. We used qualitative inquiry to assess the engagement of U.S. institutions as reported by 80 AFCs that completed a five-year cycle of participation. We conducted directed content analysis using paired AFC action plans and progress reports (n=56) and classified engagement using a priori indicators by higher educational core activities (teaching, research, and service), core phases (e.g., measure) and clustered domain areas (e.g., built environment). Results reveal engagement across all core activities of higher education with most efforts in research, in all areas of the AFC model with most reported in the action phase, and across all clustered domains of practice with the greatest amount identified in the social environment. We identify opportunities for greater engagement and leadership by higher education in our age-friendly world.

